# Supplementary material for: Comparative multiomics analysis of cell physiological state after culture in a basket bioreactor
Source: Sci Rep. 2022 Nov 23;12:20161. doi: 10.1038/s41598-022-24687-4 (PMC9686226; doi:10.1038/s41598-022-24687-4)
Supplement: Supplementary file 1 — Supplementary Information 1. [file 41598_2022_24687_MOESM1_ESM.zip › raw data/Transcriptomic raw data/6.Enrichment/2.KEGG/TE1vsTC1/UP/src/csab04210.html]

csab04210


- csab:103235922

- Up regulated genes

103235922(1.16986914788407)

- csab:103224652

- Up regulated genes

103224652(1.58918989505374)

- csab:103247197

- Up regulated genes

103247197(1.34087353024886)

- csab:103247197

- Up regulated genes

103247197(1.34087353024886)

Close
